# Spectrum of female commercial sex work in Bangui, Central African Republic

**DOI:** 10.1080/17290376.2017.1394907

**Published:** 2017-11-01

**Authors:** Jean De Dieu Longo, Marcel Mbéko Simaléko, Richard Ngbale, Gérard Grésenguet, Gilles Brücker, Laurent Bélec

**Affiliations:** ^a^ MD, MSc, PhD in Public Health, at the Centre National de Référence des Maladies Sexuellement Transmissibles et de la Thérapie Antirétrovirale, Bangui, Central African Republic.; ^b^ MD at the Service de Gynéco-obstétrique, hôpital Communautaire, Bangui, Central African Republic; ^c^ MD, MPH, PhD at the Department of Public Health, hôpital de Bicêtre, Assistance Publique-Hôpitaux de Paris, Paris Sud University, Le Kremlin-Bicêtre, France; ^d^ MD, MPH, MSc, PhD at the Laboratoire de Microbiologie, hôpital Européen Georges Pompidou, Assistance Publique-Hôpitaux de Paris, and Faculté de Médecine Paris Descartes, Université Paris Descartes (Paris V), Sorbonne Paris Cité, Paris, France

**Keywords:** female sex workers, classification, Central African Republic, risk factor, prevention programmes, Travailleuse du sexe, classification, Typologie, Républiuqe Centrafricaine, Facteur de risque, Programmes de prévention

## Abstract

Classification of professional and non-professional female sex workers (FSWs) into different categories, never previously reported in the Central African Republic (CAR), may be useful to assess the dynamics of the human immunodeficiency virus (HIV) epidemic, design operational intervention programmes to combat HIV and other sexually transmitted infections (STIs) and to adapt these programmes to the broad spectrum of sexual transactions in the CAR. Our study proposes a socio-behavioural classification of FSWs living in the CAR and engaged in transactional and commercial sex. Thus, the aims of the study were these: (i) to categorize FSWs according to socio-anthropologic criteria in Bangui and (ii) to examine the association between a selection of demographic and risk variables with the different categories of female sex work as an outcome. A cross-sectional questionnaire survey was conducted in 2013 to describe the spectrum of commercial sex work (CSW) in Bangui among 345 sexually active women having more than 2 sexual partners, other than their regular partner, during the prior 3 months and reporting to have received money or *gifts* in return for their sexual relationships. According to socio-behavioural characteristics, FSWs were classified into six different categories. Professional FSWs, constituting 32.5% of the interviewed women, were divided in two categories: *pupulenge* (13.9%), i.e., dragonflie*s* (sometimes called *gba moundjou*, meaning literally *look at the White*) consisting of roamers, who travel around the city to hotels and nightclubs seeking wealthy clients, with a preference for French men; and the category of *kata* (18.6%), i.e., FSWs working in poor neighbourhoods. Non-professional FSWs, constituting 67.5% of the interviewed women, were divided into four categories: street and market vendors (20.8%), students (19.1%), housewives (15.7%) and unskilled civil servants (11.9%). In general, CSW in the CAR presents a remarkably heterogeneous phenomenon. Risk-taking behaviour regarding STI/HIV infection appears to be different according to the different categories of female CSW. The groups of *katas* and street vendors were poorer and less educated, consumed more alcohol or other psycho-active substances (cannabis, tramadol and glue) and, consequently, were more exposed to STI. Our results emphasise the high level of vulnerability of both poor professional FSWs (*kata*) and non-professional sex workers, especially street vendors, who should be taken into account when designing prevention programmes targeting this population for STI/HIV control purposes.

## Résumé

La typologie des femmes travailleuses du sexe (TS), incluant les prostituées dites ‘professionnelles’ et celles qualifiées de ‘non professionnelles’, n'a jamais été proposée en République Centrafricaine (RCA). Une telle classification des différentes formes de prostitution peut être utile pour évaluer la dynamique de l'épidémie d'infection à VIH, et pour concevoir des programmes de prévention opérationnels contre la propagation de l'infection à VIH et les autres infections sexuellement transmissibles (IST), qui soient adaptés au large spectre des différentes formes de prostitution existant en RCA. Notre étude propose une classification socio-comportementale des TS vivant à Bangui, la capitale de la RCA. Ainsi, les buts de notre étude ont été: i) de proposer une classification typologique des TS à Bangui selon des critères socio-comportementaux et ii) d'évaluer les associations possibles entre une sélection de variables démographiques et de variables évaluant les prises de risque et les différentes catégories de prostituées. L'enquête transversale réalisée sur la base d'un questionnaire proposé par un partenaire sociocommunautaire a été réalisée en 2013 pour décrire le spectre des différentes formes de prostitution parmi 345 femmes sexuellement actives ayant eu plus de 2 partenaires sexuels autres que leur partenaire régulier durant les 3 derniers mois, et ayant reçu de l'argent ou encore un ‘cadeau’ en échange de leurs relations sexuelles. Selon leurs principales caractéristiques socio-comportemenetales, les TS ont pu être classifiées en 6 catégories. Parmi les TS qualifiées de ‘professionnelles’, constituant 32,5% des femmes interviewées, il existait deux catégories différentes: celles des ‘pupulengue’ (ou encore ‘papillons’ en Français, également dénommées ‘gba moundjou’ ce qui signifie littéralement ‘qui regarde le Blanc’) (13,9%) qui constituent des ‘trotteuses’ parcourant la ville d'hôtels en night clubs à la recherche de clients fortunés, de préférence Blancs; et la catégories des ‘katas’ (18,6%), TS pauvres œuvrant en périphérie dans les banlieues. Parmi les travailleuses du sexe qualifiées de ‘non professionnelles’, constituant 67,5% des femmes interviewées, il existait quatre catégories différentes: celle des vendeuses de fruits et légumes (20,8%), celles des étudiantes (19,1%), celles des femmes au foyer (15,7%) et enfin celles des femmes fonctionnaires (11,9%). Ainsi, d'une façon globale et remarquable, les TS en RCA représentent des populations extrêmement hétérogènes. Les prises de risque vis-à-vis de l'infection à VIH et des autres IST apparaissaient différentes selon chaque catégorie. Ainsi, le groupe des ‘katas’ et celui des vendeuses de rue étaient les plus pauvres et les moins éduqués, caractérisés par une consommation importante d'alcool ou d'autres psychotropes (comme le cannabis, le tramadol ou la colle), et montraient les prévalences de prises de risque les plus élevées vis-à-vis de l'infection à VIH. La grande vulnérabilité des TS appartenant aux groupes des ‘katas’ et des vendeuses de rue justifie pleinement des programmes d'intervention ciblant spécifiquement ces femmes à haut risque d'infection à VIH.

## Introduction

The human immunodeficiency virus (HIV) epidemic in the Central African Republic (CAR) is widespread and generalized with an overall prevalence of 4.6% throughout the country in 2011, and 6.2%, among women of childbearing age in Bangui (UNFPA, 2012; UNICEF, 2011). The principal mode of transmission is heterosexual intercourse (UNAIDS, 2012), with sexually transmitted infections (STIs) acting as major modulatory cofactors (Blankhart, Müller, Gresenguet, & Weis, 1999; Gresenguet, Belec, Martin, & Georges, 1991; Grésenguet et al., 2002; Martin P.M. et al.,^1992^; Mbopi-Kéou et al., 2000). Specifically, a strong synergistic association between heterosexual transmission and acquisition of HIV and genital herpes type 2 has been demonstrated in sexually active adults (LeGoff et al., 2007; Mbopi-Kéou et al., 2000). The acquisition of HIV through heterosexual intercourse occurs at a higher rate in exposed women than in men, resulting in high female vulnerability and HIV prevalence among young adult women (Mathiot, Lepage, Chouaib, Georges-Courbot, & Georges, 1990).

In Africa, sex work may be stigmatized and illegal, and female sex workers (FSWs) represent a particularly marginalized section of the population. Poverty, inequality and commercial sex work (CSW) are inextricably linked, with most sellers of sex being female and poor (Choi, 2011; Harcourt & Donovan, 2005; Ngugi, Roth, Mastin, Nderitu, & Yasmin, 2012). Current research on the extent and context of CSW in sub-Saharan Africa includes several important outcomes (Ngugi et al., 2012; Shannon et al., 2015). One important goal is to propose clear and differentiated definitions of sex work and transactional sex (Harcourt & Donovan, 2005; Nagot et al., 2002). Indeed, policy debates are often fuelled by passionate advocates both for and against the selling of sex. Feminist debates on the issue are fervent and often polarized, with one side arguing CSW is always forced and, thus, equates to rape (Schwitters et al., 2015; Sherwood et al., 2015); the other side views CSW as a form of work that should require policy reforms that provide better working conditions and protection for sex workers. A better understanding of CSW in Africa could help in the review and improvement of sex work legislation (Harcourt & Donovan, 2005). Finally, the categorization of FSW could help to conceive and adapt public health interventions in the direction of specific groups of vulnerable women, by improving understanding of the location, population size, density and organizational typologies of CSW (Ikpeazu et al., 2014; Kimani et al., 2013). Specific and adapted intervention may help to prevent STIs, such as HIV (MacAllister et al., 2015; Mountain et al., 2014a; Nagot et al., 2002; Scheibe, Drame, & Shannon, 2012; Vuylsteke et al., 2012) and to facilitate the proposal of antiretroviral treatment for HIV-infected FSWs with the potential impact on decreasing HIV sexual transmission (Low et al., 2015; Mountain et al., 2014b).

In the context of the extreme poverty of the CAR, female CSW constitutes, a priori, a high-risk behaviour regarding HIV infection, as generally observed in sub-Saharan Africa (Ngugi et al., 2012; Prüss-Ustün et al., 2013). However, to this point, little consideration has been given to the different patterns and faces that CSW displays in the CAR. In West Africa, Nagot and colleagues proposed a CSW classification in Burkina Faso of six different categories, including four groups of non-professional FSWs (Nagot et al., 2002). However, to the best of our knowledge, no attempt at FSW categorization has been reported in the CAR. Most previous studies focused on women who identified themselves as commercial FSWs, representing only the visible CSW network, with the nature and role of clandestine CSW being generally ignored (Nagot et al., 2002). Classification of the different FSWs operating in the CAR may be a tool to assess the dynamics of the HIV epidemic, design operational intervention programmes against HIV and other STIs and adapt these programmes for this at-risk, often neglected, vulnerable and crucial core population (Overs & Loff, 2013; Scheibe et al., 2012).

Finally, the aim of the present work is to propose a socio-behavioural classification of the various modalities of professional and non-professional transactional and commercial sex activity among adult women living in Bangui, the capital city of the CAR.

## Study population and methods

### Study design

The study was a descriptive, quantitative, population-based cross-sectional survey, using a face-to-face questionnaire to collect data in 2013 among FSW populations living in Bangui. Field survey and data collection were preceded by an exploratory survey and pilot study done to validate the instrument of data collection, especially the questionnaire used for the study. A snowball sampling ‘targeted’ was used for identifying FSW.

## Enrolment and selection criteria

An empirical purposive sampling strategy was used to select the study FSW population by which the representativeness of commercial sex work in Bangui was expected to be ensured by the reasoned approach of our enrolment and selection protocol.

In order to avoid selection bias, the vast majority of the most notorious places were sex trade occurs in Bangui, including schools and universities, were selected. Furthermore, invitations to come to the CNRMST/SIDA of Bangui to complete the questionnaires were distributed to groups of women active on commercial sites and sites suspected of sexual transactions without knowing their possibly tariffed or unpaid sexual practices.

**Fig. 1.**
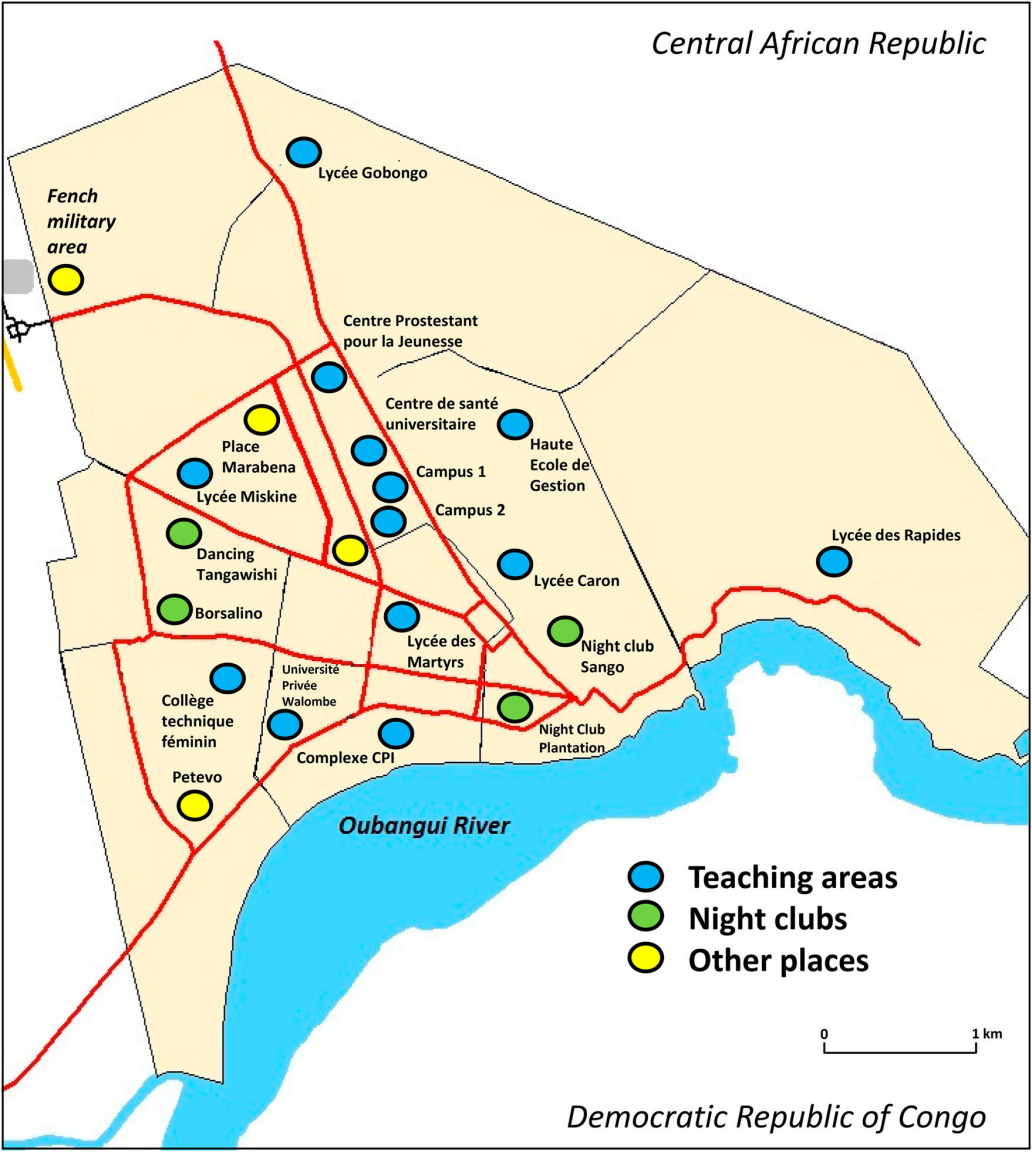
Map showing the location of inclusion study sites in Bangui, including 13 secondary schools and university attendance sites (blue round), 4 dancing places (Sango and Plantation nights clubs downtown; Tangawishi and Borsalmino dancings at periphery) (green round, and 4 other emplacements notoriously known as couples’ meeting places, such as the French military area near the airport (yellow round).

Due to a lack of information on the study target of FSWs in Bangui, our approach was to first establish a possible cartography of the vast majority of well-known areas of sex transactions throughout the city. Fig. 1 depicts the eight most notorious places in Bangui where sex trade occurs, and these places include such things as bars, formal and informal trading places, night clubs, hotels, foreign military premises and popularly visited passageways. In addition, 13 secondary schools and university institutions were also selected.

In each study enrolment area, women were recruited on a volunteer basis. Twelve peer educators contacted all women attending the 21 selected sites during a 3-month period and proposed that they be included in the study after an oral explanation and collective awareness sessions on the objectives of the survey.

After oral consent, the selected women were invited, with paid transportation, to come to the Centre National de Référence des Maladies Sexuellement Transmissibles et du SIDA (CNRMST/SIDA) of Bangui, the city’s main clinical centre for voluntary HIV counselling, testing and diagnosis and to receive care for those patients with STIs (Grésenguet et al., 2002). To avoid selection bias, invitations to come to the CNRMST/SIDA of Bangui to complete the questionnaires were distributed to groups of women active on commercial sites and sites suspected of sexual transactions without knowing their possibly tariffed or unpaid sexual practices.

The study questionnaire was developed by National programme against AIDS using standardized indicators on HIV/AIDS/STIs behavioural surveillance surveys for use with key populations, both in French and Sango, the national language. The questionnaire was administered at the CNRMST/SIDA by experienced counsellors specifically trained for this purpose. It contained questions on demographic and behavioural data, knowledge of HIV infection, associated diseases and comorbidities, and possible sex transactions, and included the following sections: (0) identification data (5 items), (1) background characteristics (12 items), (2) marriage, family, work (9 items), (3) sexual history: numbers and types of partners (3 items), (4) sexual history: paying clients (6 items), (5) sexual history: non-paying partners (6 items), (6) use of male and female condoms (7 items), (7) STIs (5 items), (8) knowledge, opinions and attitudes towards HIV and AIDS (18 items) and (9) STIs and HIV prevention (3 items). In addition to the questionnaire, open questions were asked about the characteristics of sex work by the counsellors.

All women attending the CNRMST/SIDA benefited from free HIV testing, gynaecological examination of STIs, laboratory analysis where necessary and appropriate treatment for those suffering from genital or HIV infections. It is worth noting that the invitation to attend the clinical centre was proposed to all women, regardless of possible paid sexual practices. Note that the study participants were not recruited because of external evidence of sex work, such as evocating clothing or dressing, nor because obvious lingering around the sex trade hotspot.

Inclusion criteria consisted of the following: being a volunteer, having given oral consent, being sexually active, having more than three sexual partners, other than a regular partner, during the prior three months and having received money or gifts in return for sexual relationships. Exclusion criteria included not willing to participate to the study, having had a sexual relationship to obtain a job or to obtain a good average at school or university. All women who reported having more than three sexual partners other than their regular partner during the last three months and reporting having received money or ‘gifts’ in return of sexual relationships were finally included for study analysis.

Finally, a total of 1384 women from inclusion sites in the capital city of Bangui attended the CNRMST/SIDA from March to August 2013. All women attending benefited from clinical services (clinical examination, adapted biological analyses and care), received an information session on HIV and STIs, and completed a face-to-face questionnaire.

## Study analysis

The following variables were taken into consideration. The variable ‘FSW’ was defined as ‘having more than 2 sexual partners other than the regular partner during the prior three months and having received money or “gifts” in return for sexual relationships’. The variable ‘professional FSW’ was defined as ‘self-report of main resources from paid sexual transactions’. The variable ‘non-professional FSW’ included women who did not identify themselves as sex workers, reporting another activity as their main source of income or were still secondary or university students, but who nevertheless had sexual transactions during the prior three months and reported having at least two sexual partners outside their regular partner in this period. The variable ‘women having a good knowledge on HIV infection’ included women knowing that consistent condom use was the most effective way to avoid HIV infection and that even a healthy looking person can be infected with HIV. The variable ‘high risk for acquiring HIV infection’ characterized women reporting their first sexual intercourse at younger than 15 years of age, an almost daily consumption of more than 3 glasses of alcohol, use of drugs or psycho-active substances, non-use of condoms during sexual intercourse with (vaginal or anal) penetration in the prior 3 months with a casual partner and a past history of STIs in the prior 3 months.

The results of the previously described variables of the questionnaire were entered into an Excel sheet and analysed using Epi Info^TM^ version 3.5.1 (Center for Disease Control and Prevention, Atlanta, GA, USA). Statistical analyses were conducted using Epi Info™ 7 software (CDC, Atlanta, Georgia, USA). *P*-values were calculated using Pearson’s *χ* ^2^ test or Fisher’s exact test for categorical variables and the non-parametric Mann–Whitney *U*-test for non-categorical variables in order to compare differences in socio-demographic characteristics according to socio-behavioral categories.

## Ethical approval

The study was formally approved on by the Scientific Committee Faculty of Health Sciences of Bangui (‘Comité Scientifique Chargé de la Validation des Protocoles d’Etudes et des Résultats’/‘CSCVPER’) (agreement UB/FACSS/CSCVPER), constituting the National Ethical Committee. All included women gave their informed oral consent to participate in the study. No consent from the parents or guardians of minor women could be obtained. For each included woman, the record of the consent to participate in the study was documented on each questionnaire. This consent procedure was formally approved by the National Ethical Committee.

## Results

### Study population

**Fig. 2.**
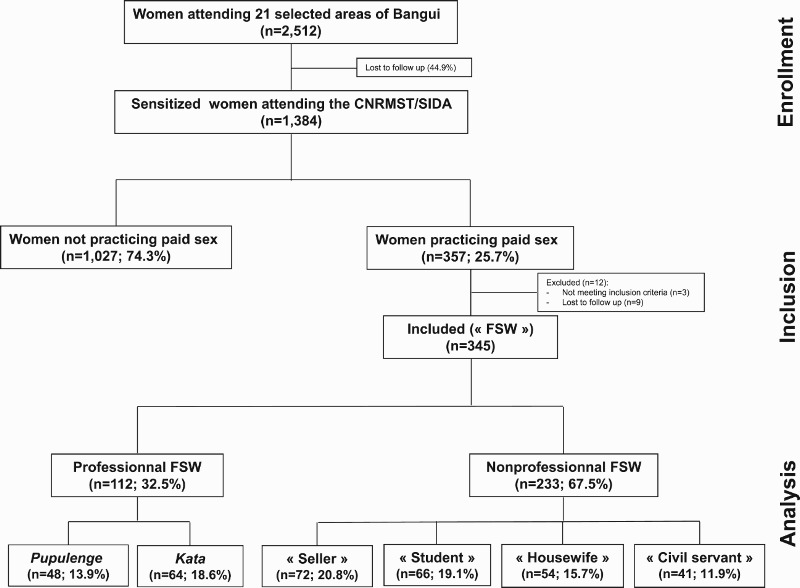
Flow diagram showing enrolment, inclusion and data analysis for the study. A total of 2512 women attending 21 areas of couples’ meeting places, including 13 secondary schools and university places, were sensitized to the study purposes and proposed to attend the ‘Centre National de Référence des Maladies Sexuellement Transmissibles et du SIDA’ (CNRMST/SIDA), the main clinic for sexually transmitted infections in Bangui; 1384 (55.1%) of them thereafter voluntarily consulted with the CNRMST/SIDA for participating to the study; 357 (35.7%) sexually active women declared having more than 2 sexual partners, other than their regular partner, during the prior 3 months and having received money as their job or gifts in return for their sexual relationships (exclusive of a job), and were included as female sex workers (FSWs) for answering a face-to-face, structured sociodemographic and behavioural questionnaire. The group of professional FSWs included women declaring paid sex as their principal sources of income, and the other women who practiced occasional paid sex and had not declared themselves as FSWs were classified as non-professional or clandestine FSWs.

A total of 2512 women received information about the objectives of the study and advice on sexual and reproductive health, as presented in the study flow chart (Fig. 2). A subgroup of 1662 women was recruited in 8 notorious pickup places, and another 850 in secondary schools (*n* = 399) and university institutions (*n* = 451). Thousand hundred and twenty-eight (44.9%) were lost to follow-up. Among the women sensitized to the study purposes, 1384 (55.1%) attended the CNRMST/SIDA, and 357 (25.7%) reported gainful compensation for sex within the prior 3 months and as well as having at least 3 sex partners during the prior 3 months. However, 12 questionnaires were excluded because 3 were incomplete and 9 were abandoned during execution. Finally, 345 FSW questionnaires were selected for study analysis. Among them, 112 (32.5%) women reported regular paid sex transactions (commercial) as their main source of income, identified themselves as FSWs, and were classified as professional FSWs. The remaining 233 (77.5%) women reported another activity as their main source of income (*n* = 167) or were still schoolgirls/students (*n* = 66), but, nevertheless, entertained commercial sex transactions over a recent period (prior 3 months) with more than 2 sex partners, apart from their regular partner, and, thus, were classified as non-professional FSWs.

### Categorization of commercial sex work

Besides the professional sex workers, non-professional sex workers appeared clearly as a frequent secondary population of FSWs. We attempted to define socio-behavioural categories, encompassing this large spectrum (professionals and non-professionals) of commercial sex activity in two different ways, depending on the main social and behavioural characteristics highlighted through the questionnaire.

Firstly, we classified *official* professional FSWs (32.5%) according to their work site. Indeed, there was a marked separation between the two categories of professional FSWs, depending on whether they worked downtown, near hotels, in bars and night clubs or in the peripheral areas of Bangui.

The first category of professional FSWs (13.9%) included the *pupulenge*, a Sango word meaning dragonfly (also called *gba moundjou*, a black woman who has sex with white men, i.e., a high-class FSW).

The second category of professional FSWs included women working in the poor neighbourhoods of Bangui, who are called *kata* (a pejorative word with no other Sango meaning).

Secondly, we classified non-professional FSWs (67.5%) according to their reported main activity. Thus, four categories of non-professional FSW were identified according to their occupation as street vendors, schoolgirl or student, housewife or unskilled civil servant.

The first category of non-professional FSWs included market and street vendors (20.8%) consisting of mobile women selling fruits or vegetables along the main roads of the city by carrying a tray on their heads. Actually, this activity often hides a commercial sex business. The second category was schoolgirls or students (19.1%) involved in occasional transactional sex, particularly during holidays. Most do so to pay for their school fees or for their living expenses, but others want money to buy clothing or jewellery.

The third category of non-professional FSWs consisted of housewives (15.7%).

Finally, the fourth category of non-professional FSWs was made up of unskilled female civil servants (11.9%) working as soldiers or in the public service.

### Socio-demographic characteristics of the FSW population

Table 1 depicts the characteristics of the 345 FSW selected for study analysis. Their mean age was 23.5 years, with a median age of 23.0 years (range, 14–47 years). In 27% of the cases, the FSWs were younger than 20 years of age. The majority of FSWs originated from the CAR (90%), and, to a lesser extent, from neighbouring countries including the Democratic Republic of Congo (6%) and other countries (4%) (Congo Brazzaville, Chad and Cameroon). Most FSWs (69%) had a secondary-level education, equally distributed between college and high school. Fewer women had a higher education level (16%); only a minority of FSW had a low level of education (15%). The mean age of first sexual intercourse was 17 years (range, 10–24 years).

**Table 1.** Characteristics of 345 included women practicing paid sex and living in Bangui.

	Category of female sex workers						P*
	Professional (*n* = 112)		Nonprofessional (*n* = 233)				
	*Pupulenge* (*n* = 48)	*Kata* (*n* = 64)	Seller (*n* = 72)	Student (*n* = 66)	Housewife (*n* = 54)	Civil servant (*n* = 41)	
*Age*							
Median (years)	21	20	25	20	27	27	
Range	[16–29]	[14–36]	[14–38]	[16–28]	[18–46]	[22–47]	–
Less than 18-year-old (%)	21	14	14	17	2	0	
*Nationality*							
Central African Republic	39 (81%)	50 (78%)	68 (94%)	62 (94%)	51 (94%)	41 (100%)	
Democratic Republic of Congo	6 (13%)	8 (13%)	2 (3%)	2 (3%)	2 (4%)	0 (0%)	NS
Other	3 (6%)	6 (9%)	2 (3%)	2 (3%)	1 (2%)	0 (0%)	
*Education level*							
Illiterate	0 (0%)	37 (58%)	32 (44%)	0 (0%)	6 (11%)	0 (0%)	<0.05
Secondary level	39 (81%)	23 (36%)	31 (43%)	30 (46%)	27 (50%)	1 (2%)	
Superior level	9 (19%)	4 (6%)	9 (13%)	36 (55%)	21 (39%)	40 (98%)	
*Matrimonial status*							
Unmarried	45 (94%)	64 (100%)	59 (82%)	54 (82%)	22 (41%)	20 (49%)	<0.05
Married	03 (6%)	0 (0%)	13 (18%)	12 (18%)	32 (59%)	21 (51%)	
*Number of children*							
0	22 (46%)	44 (69%)	15 (21%)	42 (64%)	4 (7%)	4 (10%)	<0.05
1–2	26 (54%)	20 (31%)	52 (72%)	24 (36%)	35 (65%)	33 (80%)	
More than 3	0 (0%)	0 (0%)	5 (7%)	0 (0%)	15 (28%)	4 (10%)	
*Knowledge on HIV/AIDS*							
Weak	3 (6%)	14(22%)	45 (63%)	1 (2%)	9 (17%)	2 (5%)	<0.05
Middle	8 (17%)	21 (33%)	18 (25%)	5 (7%)	9 (17%)	3 (7%)	
Satisfactory	37 (77%)	29 (45%)	9 (12%)	60 (91%)	36 (66%)	36 (88%)	
*Self-assessment of HIV risk*							
Yes	40 (83%)	24 (37%)	12 (17%)	35 (53%)	30 (56%)	13 (32%)	<0.05
No	8 (17%)	40 (63%)	60 (83%)	31 (47%)	24 (44%)	28 (68%)	
*Knowledge on HIV serostatus*							
Yes	36 (75%)	13 (20%)	20 (28%)	44 (67%)	24 (44%)	25 (61%)	<0.05
No	12 (25%)	51 (80%)	52 (72%)	22 (33%)	30 (56%)	16 (39%)	
*Past history of STI*							
Yes	9 (18%)	24 (38%)	31 (43%)	10 (15%)	15 (28%)	6 (15%)	<0.05
No	39 (81%)	40 (62%)	41 (57%)	56 (85%)	39 (72%)	35 (85%)	
*Age of first sexual intercourse*							
10–15 years	27 (56%)	43 (67%)	39 (54%)	26 (39%)	27 (50%)	13 (32%)	<0.05
16–20 years	21 (44%)	21 (33%)	33 (46%)	40 (61%)	27 (50%)	28 (68 %)	
*Duration of prostitution*							
<1 year	8 (17%)	13 (20%)	NA	NA	NA	NA	
2–5 years	23(48%)	43 (67%)	NA	NA	NA	NA	
>5 years	17(35%)	8 (12%)	NA	NA	NA	NA	
*Condom use in last 3 months*							
No or rarely	12 (25%)	60 (94%)	61 (85%)	30 (45%)	40 (74%)	18 (44%)	<0.05
Always	36 (75%)	4 (6%)	11 (15%)	36 (56%)	14 (26%)	23 (56%)	
*Condom use in last week*							
No or rarely	0 (0%)	40 (62%)	48 (67%)	12 (18%)	29 (54%)	11 (27%)	<0.05
Always	48 (100%)	24 (38%)	24 (33%)	54 (82%)	25 (46%)	30 (73%)	
*Alcohol consumption*							
No or 1 fold per week	43 (90%)	8 (13%)	37 (51%)	50 (76%)	27 (50%)	33 (80%)	<0.05
Every day	5 (10%)	56 (87%)	35 (49%)	16 (24%)	27 (50%)	8 (20%)	
*Cannabis consumption*							
No or 1 fold per week	47 (98%)	55 (86%)	71 (99%)	66 (100%)	54 (100%)	41 (100%)	<0.05
Every day	1 (2%)	9 (14%)	1 (1%)	0 (0%)	0 (0%)	0 (0%)	
*Glue inhalation*							
No or 1 per week	48 (100%)	53 (83%)	72 (100%)	65 (98%)	54 (100%)	41 (100%)	<0.05
Every day	0 (0%)	11 (17%)	0 (0%)	1 (2%)	0 (0%)	0 (0%)	

Notes: STI: Sexually transmitted infection; NA: Not attributable; NS: Not significant.

*χ ^2^ test; *P* < .05 is considered as significant.

Our first approach for analysis was to compare professional versus non-professional FSWs. Professional FSWs median age of 21 years (range, 14–36 years)] were younger than non-professional FSWs [median age 25 (range, 14–47 years)] (*P* < .05); 30% of professional FSW were younger than 18 years of age versus 15% of non-professional FSWs (*P* < .05). The proportion of foreigners was three times higher among non-professional FSWs than among professional FSWs (17.1% versus 5.7%; *P* < .05). Professional FSWs showed slightly higher levels of education than non-professional FSWs, achieving a level of secondary education of 55% versus 38% (*P* < .05). The percentage of FSWs showing satisfactory knowledge of HIV/AIDS was similar among professional (59%) and non-professional (48%) groups; however, the percentage of erroneous knowledge of HIV/AIDS appeared to be higher among clandestine FSWs (76%) than official FSWs (57%) (*P* < .001). The proportion of knowledge of HIV serological status was 56% in professional FSWs and 52% in non-professional FSWs (NSD). A past history of STIs was noted in 30% of professional FSWs and in 27% of non-professional FSWs (NSD). The median age of first sexual intercourse was younger in the professional group [median age 16 years (range, 10–19 years)] than in non-professional group [median age 17 years (range, 11–24 years)] FSW (*P* < .01), and the proportion of women reporting a sexual experience before the age of 15 was higher in the professional group (63%) than in the non-professional group (47%) of FSWs (*P* < .01). The percentage of FSWs with a self-perception of high HIV risk during the last sexual intercourse was higher in professional FSWs (61%) than non-professional FSWs (43%) (*P* < .001). The proportion of women who proposed a condom to their male sexual partner during the last sexual intercourse, and the proportion of those who effectively used a condom during the last sexual intercourse were similar in professional and non-professional FSWs, 17% versus 19% and 57% versus 64%, respectively (NSD). The percentage of women reporting regular (daily) consumption of alcohol was higher in official FSWs (55% consuming more than three 65 cl bottles of beer; three standard servings of alcohol) than clandestine FSWs (37% consuming more than three 65 cl bottles of beer (*P* < .002).

Regular (daily) consumption of psycho-active substances (cannabis, tramadol and glue) was exclusively observed among professional FSWs (82%) versus (9%) non-professional FSWs, *P* < .001).

Taken together, these observations demonstrate similar characteristics, but also demonstrate seemingly different profiles among professional and non-professional FSWs (Table 2). Thus, professional FSWs and non-professional FSWs have in common similar proportions of knowledge of HIV their serological status, past history of STIs and capability of proposing a condom to their last male sexual partner or to effectively use a condom during the last sexual intercourse. Regarding their differences, professional FSWs had generally reached legal majority age, but one-third of them were younger; about one out of five were foreigners from neighbouring countries; they were often relatively well educated; they had begun sexual life early; their knowledge of HIV was often satisfactory, without erroneous knowledge or misconception on AIDS, and they had a high proportion of self-perception of a high risk for HIV. Finally, most professional FSWs regularly consumed alcohol or, to a lesser extent, psycho-active substances. Non-professional FSWs were older; they generally originated from the CAR; they had their first sexual intercourse near the age of majority; they were often poorly educated; their knowledge of HIV was often satisfactory, but they may have had erroneous information about AIDS; and they showed a low proportion of self-perception of the high risk of HIV. Only a minority regularly consumed alcohol, and none of them consumed psycho-active substances.

**Table 2.** Principal similarities and differences characterizing the two main categories of female sex workers (FSW) living in Bangui, the capital city of the Central African Republic, according to recognition regarding having had paid sex during the last three months in order to obtain economic compensation with more than three sexual partners.

	Categories	
	Self-declared FSW	Clandestine FSW
Similarities	Same proportion of known past history of sexually transmitted infections Knowledge of HIV serological status Negotiation capabilities during paid sex	
Differences	Professional Principal source of income Median age: 21 years 20% foreigner Generally well educated (except the ‘kata’) Young entry in sexuality Satisfactory knowledge on HIV/AIDS with high perception of high risk for HIV Frequent regular alcohol or psycho-active substances consumption	Nonprofessional Secondary source of income Median age: 25 years Rarely foreigner (90% from CAR) Poorly educated (in particular the ‘vendors’) Entry in sexuality near the age of majority Frequent erroneous knowledge on AIDS with low perception of risk for HIV Infrequent alcohol or psycho-active substance consumption

Our second approach for analysis was to compare professional FSWs, the *pupulenge* and *kata* subgroups (Tables 1 and 3). *Kata* were slightly younger than *pupulenge* (*P* < .05). The level of education was significantly lower among *kata* (*P* < .001). The number of children was higher among *pupulenge* than *kata* (*P* < .02). The percentage of FSWs with a self-perception of high HIV risk during the last sexual intercourse was much higher among *pupulenge* (83%) than among *kata* (37%) (*P* < .001). The vast majority of *pupulenge*, in contrast to a minority of *kata,* knew their HIV status (*P* < .001). The proportion of women who proposed a condom to their male sexual partner during the last sexual intercourse and the proportion of those who effectively used a condom during the last sexual intercourse were much higher among *pupulenge* than *kata* [(75% versus 6% (*P* > .001), and 100% versus 38% (*P* < .001)]. The *kata* consumed more alcohol and more psycho-active substances than the *pupulenge* (*P* < .001). Combined, the category of official FSW comprised two subgroups of FSWs that presented fewer similarities than differences. The similarities were that they both basically considered themselves as FSWs, originating from the CAR, remaining largely *free* without a stable partner and, most often, having no or few children. The differences were more evident. The *pupulenge* were relatively well educated, exclusively worked in places with a certain reputation downtown, such as bars or international hotels and night clubs, where they occasionally consumed alcohol, mainly beer, and, above all, where they could have contact with French men, military officers, rich foreigners or travellers; their knowledge of HIV/AIDS was thorough, including knowledge of their own HIV status with a high perception of being at risk for HIV, and their capabilities to negotiate safe sex were also elevated, with nearly systematic use of condom during paid sex. In contrast, the *kata* were less educated, worked in the remote, poor and popular suburbs of Bangui, along the streets, reaching out to local men living in the same neighbourhoods, whatever their origin, had a poor knowledge of HIV/AIDS and of their own HIV status and on their own risk of exposure to HIV, with a weak ability to negotiate protection during paid sex, in a context of nearly systematic consumption of alcohol and other psycho-active substances.

**Table 3.** Principal similarities and differences characterizing the so-called ‘pupulengue’ and ‘kata’ subgroups of professional female sex workers living in Bangui.

	Subgroups	
	‘Pupulengue’	‘Kata’
Similarities	Self-declared prostitutes Originating from the CAR Unmarried or ‘free girl’ No or few children Same proportion of known past history of sexually transmitted infections Similar age of entry in sexuality Similar duration of prostitution	
Differences	Work exclusively downtown Preference for White men or foreigners Median age : 21 years Well educated >50%: 1–2 children High perception of high risk for HIV Frequent knowledge of HIV status High level of self-protection during paid sex Strong negotiation capabilities during paid sex Occasional alcohol consumption Infrequent psycho-active substances consumption	Work in poor suburbs of Bangui Clients usually Black men without preference on their nationalities (men from CAR as well as foreigners) Median age: 20 years Poorly educated #70%: no child Low perception of risk for HIV Infrequent knowledge of HIV status Low level of self-protection during paid sex Weak negotiation capabilities during paid sex Daily alcohol consumption Frequent psycho-active substances consumption

Our third analysis approach was to compare the four discrete groups among non-professional FSWs (Table 4). Non-professional FSWs practiced occasional paid sex as a secondary source of income, but did not consider or report themselves as FSWs. They were most often originating from the CAR. Schoolgirls and students were the youngest, with a median age of 20 years, and housewives and civil servants were the oldest, with a median age of 27 years (*P* < .01). The level of education among the four subgroups was heterogeneous, with civil servants showing a generally higher level, followed by schoolgirls/students, housewives and street vendors who were the least educated subgroup with nearly half being illiterate (*P* < .05). Most housewives and civil servants were married, while most street vendors and schoolgirls/students were single (*P* < .01). Housewives had the highest number of children, followed by civil servants and street vendors, with schoolgirls/students who generally had no children (*P* < .01). Knowledge of HIV/AIDS was satisfactory among most schoolgirls/students, followed by civil servants and housewives, but it was poor or very poor among street vendors (*P* > .01). About half of housewives and schoolgirls/students perceived themselves as at-risk for HIV, compared to only one-third of civil servants and only one of five street vendors (*P* < .01). Schoolgirls/students and civil servants generally knew their HIV status, in contrast to a minority of housewives and only one-third of street vendors (*P* < .01). A past history of STIs was seen most frequently in street vendors and in one-third of housewives (*P* < .05). Most schoolgirls/students and civil servants used condoms regularly, including during the last paid sexual intercourse, while only a minority of housewives and even a lower number of street vendors used them (*P* < .005). Alcohol was consumed by around half of the housewives and street vendors and one-quarter of students and civil servants (*P* < .01). Taken together, the four subgroups of non-professional FSWs appeared very heterogeneous. The only similarities were that they originated from CAR, had begun sexual life at a similar age and did not use psycho-active substances. The subgroup of street vendors appeared to be the most vulnerable, with poor education, little knowledge of HIV/AIDS, frequent past history of STI, weak capabilities to negotiate safe sex and frequent consumption of alcohol. The three other groups showed more subtle differences, notably as regards marital status, number of children, past history of STI and alcohol consumption. They were well educated and had a good knowledge of HIV/AIDS and a good perception of their own HIV risk. Apart from housewives, they frequently used condoms and showed high capabilities to negotiate save sex.

**Table 4.** Principal similarities and differences characterizing the four main subgroups of nonprofessional, clandestine female sex workers living in Bangui.

	Subgroups			
	Seller	Schoolgirl/student	Housewife	Civil servant
Similarities	Occasional paid sex as secondary source of income in women not feeling themselves as prostitutes Originating from the CAR Similar age of entry in sexuality No consumption of psycho-active substances			
Differences	Median age : 25 years Poor educated Generally single #70%: 1–2 children Poor knowledge on HIV Low perception of HIV risk Infrequent knowledge of HIV status Frequent past history of STI Rarely condom use #50%: alcohol	Median age : 20 years Well educated Generally single >60%: no child Good knowledge on HIV Good perception of HIV risk Frequent knowledge of HIV status Infrequent past history of STI Frequent condom use #25%: alcohol	Median age : 27 years Well educated Generally married #90%: 1–3 children Good knowledge on HIV Good perception of HIV risk Middle knowledge of HIV status Frequent past history of STI Infrequent condom use 50%: alcohol	Median age : 27 years Highly educated Generally married #80%: 1–2 children Good knowledge on HIV Middle perception of HIV risk Frequent knowledge of HIV status Infrequent past history of STI Frequent condom use 20%: alcohol

## Discussion

Female CSW until now has not been sufficiently investigated in the CAR, although it is readily acknowledged to be highly prevalent (Grésenguet et al., 2002; Mbopi-Kéou et al., 2000). Thus, we herein conducted a cross-sectional survey in a very heterogeneous population of women involved in commercial sex transactions. We described a large socio-behavioural spectrum of commercial sex activities comprising both professional and non-professional FSWs living in Bangui, the capital city of the CAR. We first observed that an unexpectedly high proportion of women involved in CSW do not identify themselves as FSWs, despite an excessively high risk of exposure. The category of non-professional FSW appears to be very heterogeneous including four subgroups (street vendors, schoolgirls/students, housewives and civil servants). Indeed, transactional sex relationships in which the participants frame themselves not in terms of FSW/clients, but rather as girlfriends/boyfriends, are very common and widespread in Bangui, likely constituting the foundation of paid sex. The deep sociological and cultural background, including profound gender inequalities, as well as the practices of polygamy and of male monetary compensation for female sex, may explain the importance of these non-professional groups, accentuated by the current difficult socio-economic conditions. In addition, about one-third of FSWs declare themselves as professional FSWs, with two groups of women involved. This kind of CSW is well known in Bangui, as shown by the popular local language opposing FSWs for wealthy men, mainly white men, *pupulenge,* and FSWs opposing for local men at the grassroots level, kata, representing the two subgroups of FSWs who are quite different in regard to their customers, work places, levels of education, risk-taking vis-à-vis HIV infection and consumption of alcohol and psycho-active substances.

Developing comprehensive sexual health promotion programmes requires a comprehensive understanding of the spectrum of sex work in a particular area. Commercial sex activity is characterized by its considerable heterogeneity. Harcourt and Donovan identified at least 25 types of sex work according to work site, the principal mode of soliciting clients or sexual practices (Harcourt & Donovan, 2005). These types of work are often grouped under the headings of *direct* and *indirect* CSW, with the latter group less likely to be perceived or to perceive themselves as sex workers. Direct CSW includes a variety of sex-related services for which the primary purpose is the exchange of sex for a fee. Other authors proposed a dual typology separating *professional* commercial sex workers from *non-professional* sex workers, to distinguish those that make this activity a primary occupation or source of income from those who practice it secondarily (Nagot et al., 2002). Finally, some authors distinguish *exposed* FSWs displaying themselves along streets, bars, hotels and trick rooms, from the others, called *clandestine* FSWs (Ahoyo, Alary, Ndour, Labbé, & Ahoussinou, 2009; Mboussou et al., 2012). In our study, we were able to classify the women in two categories on the basis on their being reported as sex workers, corresponding to professional FSW or non-professional FSW. Both categories are associated with a great variety in the social context and the possible harms associated with such paid sex transactions. The public health implications of each category may vary widely, and our study provides relevant information for conceiving and developing appropriate and targeted programmes.

We found a significant proportion of women who did not report sex work as their main activity and their main source of income or who were still schoolgirls/students, but who nevertheless had transactional sex. The category of non-professional FSW could be sub-divided into four subgroups, thus, it is widely heterogeneous. Such considerable heterogeneity among FSWs geographically, and in terms of operational typology, is well recognized (Blanchard, Khan, & Bokhari, 2008). Clandestine CSW was previously formally reported in Burkina Faso (Nagot et al., 2002) and Congo (Mboussou et al., 2012), and it is likely very common in sub-Saharan Africa. In Burkina Faso, Nagot and colleagues described four categories quite similar to those found in Bangui, including bar waitresses, street vendors, cabarets and students (Nagot et al., 2002). Clandestine commercial sex is considered to present similar sexual risks as official CSW, but also greater vulnerabilities because of the intrinsic difficulty to propose an adapted intervention programme (Ferguson & Morris, 2007). Indeed, this category of FSW is generally not targeted under national HIV and STIs control programmes (Ahoyo et al., 2009; Mboussou et al., 2012). Clandestine FSWs are more difficult to reach, engage and retain in intervention programmes (Berthé et al., 2008). Finally, clandestine FSWs are frequently more infected by HIV than official FSWs because of their low use of condoms (Berthé et al., 2008).

The two subgroups of professional FSWs, *pupulenge* and *kata*, were previously described in Ghana (Decosas, 1996), Senegal (Werner, 1993) and Burkina Faso (Nagot et al., 2002). The subgroup of women who sit on their stools and expect men in front of their working chamber, as previously reported in Burkina Faso (Nagot et al., 2002) could not be identified in our study population as apparently this does not exist in the CAR. In addition to the concept of *free girl* that applies to all, the Sango language assesses the existence of two groups of FSWs. Indeed, the term of *pupulenge* specifically designates young women seeking wealthy men (especially the French soldiers stationed in Bangui). Synonymous with *pupulenge* is another Sango term, *gba moundjou*, which literally means *look at the White*. The *pupulenge* are contacted by their customers via the telephone, sometimes via the Internet, through the hotel staff, the staff employed in the military camps (French or African peacekeeping forces), and sexual interactions take place in hotels or in private homes (escorts). They are also sought by their clients in places such as nightclubs, terraces, bars, hotels, airport arrival areas, swimming pools and gyms. The deprecating term *kata* designates a more heterogeneous population of usually young and frequently unschooled girls, living in the poor suburbs of Bangui who roam the streets of popular neighbourhoods looking for men. The *kata* seek their customers along the sidewalks or roads. They also meet with their clients in drinking establishments (clubs, bars or dance halls) of the lively and popular areas of the city. They also perform at bus stations and taxi and bus parking areas. Sexual interactions may take place in cheap hostels, similar to brothels, or even outdoors in dark places.

In this study, the risk of HIV exposure of women involved in commercial sex was found to be particularly high. The risk was associated with high frequency of unprotected sexual intercourse, history of STIs and, finally, frequent consumption of alcohol and/or psycho-active substances. Thus, 64% of each group, FSWs reported having had unprotected sexual intercourse with their last occasional sexual male partner. Unexpectedly, 94%, 85% and 74% of women belonging to the subgroups of *kata*, street vendors and housewives, respectively, reported having had the last sexual intercourse with their clients without the use of a condom. Similar observations of high-risk commercial sex have been previously reported in sub-Saharan Africa. For example, in the Republic of Congo (Brazzaville), during the 30 day period prior to being questioned, 67.0% of sex workers had sexual intercourse without condom with a client, versus 39.4% with a non-paying partner (Mboussou et al., 2012). In the Democratic Republic of Congo, however, 40% of the commercial sex workers used condoms consistently, and this pattern differed according to the category of sex partners (61.4% in the case of paying partners, versus 38.2% in the case of non-paying partners) (Kayembe et al., 2008). Taken together, these observations clearly demonstrate that the risk perception according to the category of male sex partner is variously appreciated among the population of FSWs, and, furthermore, it changes over time and with intervention programmes (Ghys et al., 2002). It is remarkable that the risk perception with paying partners was particularly low in our study population, a finding that should be taken into account when planning interventions targeting FSWs in the CAR.

The ability to negotiate condom use with sexual partners, other than their regular partner, voluntary counselling and testing should clearly be improved because they are likely to have an impact on behaviour. Another important observation is the lower frequency of condom use in the last sexual intercourse in non-professional FSWs, as compared with professional FSWs (52.7% versus 35.7%), as previously reported in the Republic of Congo (Mboussou et al., 2012). Social anthropologists have concluded that one problem in combating official or professional CSW is the development of clandestine commercial sex, which is more dangerous, firstly for its practitioners, who are harder to reach with messages about HIV, and, thus, do not change their behaviour; secondly, for their sex partners who do not systematically use condoms; and, finally, for society as a whole, to the extent that social actors are embedded in a more or less extensive informal network of sex partners (Berthé et al., 2008). A previous history of STIs, which constitutes a well identified cofactor for HIV acquisition (Laga et al., 1994) and is presented frequently as multiple co-infections (Mgone et al., 2002), was reported in 27.6% of the study’s women, and was highest in the professional subgroup of *kata* (38%) and in the non-professional subgroup of street vendors (43%). The professional subgroups of *kata* and the non-professional subgroup of street vendors are often difficult to distinguish. Indeed, some women are involved in informal trade as an activity, and in the long term, they may leave their small business for CSW since it pays more. Unexpectedly, no clandestine FSWs working in cabarets or in bars as waitresses could be observed in the present study, as described by other authors in West Africa (Ahoyo et al., 2009; Nagot et al., 2002).

Finally, the consumption of psycho-active substances, as an important cofactor contributing to sexual risk behaviours for HIV infection (Bryant, 2006), was frequent, reaching daily excessive (i.e. more than three 66 cl bottles of beer) alcohol intake in 54.5%, 44.9% and 24.2% of professional FSWs, civil servant and schoolgirl/students, respectively. It is well recognized in the context of sub-Saharan Africa that excessive consumption of alcohol promotes vulnerability and exposure of HIV and other STIs (Bryant, 2006; Weiser et al., 2006). Alcohol consumption is also a factor contributing to unprotected sex in female FSWs (Mgone et al., 2002; Rehm, Shield, Joharchi, & Shuper, 2012).

Indeed, several studies on alcohol consumption and risky sexual behaviour vis-à-vis HIV infection showed that individuals under the influence of alcohol are less likely to negotiate condom use with their casual sexual partners (Ashley, Levine, & Needle, 2006; Cooper, 2002; Rehm et al., 2012). An additional aggravating feature is that the vast majority (79.4%) of FSWs have designated drinking places as their favourites for encounters and sexual transactions. Finally, the consumption of other psycho-active substances, frequently observed among the *kata* subgroup, also increases the risk of acquiring HIV among sex workers (Fritz et al., 2001; World Health Organization, 2005). The World Health Organization recommends that information campaigns for the prevention of HIV and other STIs for general adult populations include the problem of sexual risk behaviour changes secondary to alcoholism and the consumption of other psycho-active substances, especially for vulnerable populations including sex workers and youths (World Health Organization, 2005).

Our study has some limitations. First, the study inclusions were initially carried out by random sampling stratified by type of dating sites and/or suspected commercial sex transactions. Thus, the representativeness of the included study population likely depends on the completeness of the mapping sites of suspected sexual transactions that were used as a sampling frame. Second, participants were included on a voluntary basis for completion of the questionnaire. This latter approach may be a source of recruitment bias. Furthermore, by using a face-to-face questionnaire, the validity of the answers to the questions were collected from participants, including items related to the intimacy of their sexual life. In the CAR, a number of women are unable to read and write properly, and so a self-administered questionnaire was not relevant for this reason; however, it has been documented that questions about personal privacy are best collected anonymously by means of a self-administered questionnaire (Teunis, 2001). In addition, the recruitment based on associative networks and peer educators, which constituted the only way to easily connect with the social world of commercial sex workers, may have introduced a selection bias, at least because the study participants could themselves be part of the networks of peer educators. Finally, the study respondents said they agreed to participate in the study because they considered that confidentiality was guaranteed at CNRMST/SIDA of Bangui, which is considered by the public as the benchmark of good practice in this area and which engages highly respected peer educators (Grésenguet et al., 2002). Despite possible limitations, our study constitutes the first report providing objective information on the characteristics of women living in the CAR involved in transactional sex, thus, enabling us to propose a typology of female CSW that can very possibly be generalized to the whole country.

## Conclusion and perspectives

This study’s findings indicate that female CSW in the CAR is remarkably diverse and heterogeneous, as previously pointed in the various contexts of commercial sex (Choi, 2011; Mahdavi, 2010). It seems clear that FSWs of all categories are vulnerable because of the unequal gender relationships, gender violence and the very slipperiness of the lack of criminalization of sex work in Africa. Risk-taking, vis-à-vis HIV infection, may be different according to the type and nature of female CSW in the CAR.

Our study constitutes the starting point for a reflection on the management of FSW in the CAR, including health authorities, non-governmental organizations of people living with HIV and public health researchers (mainly from the faculty of medicine). The identification of several categories of FSW will help to determine the hot spots of some forms of CSW across the country, and also to estimate the number of FSWs to be targeted. Pilot interventions should be conceived, developed in existing health care facilities, such as STI clinics like the CNRMST/SIDA of Bangui, and, thereafter, evaluated. Targeted interventions that aim to increase condom use and reduce transmission of STI and HIV infection among FSWs and their clients have been shown to be feasible and effective (Ghys et al., 2002). In addition, population-specific minimum packages of services will be defined for each category of reachable FSWs, including behaviour change, communication and condom promotion, by and in collaboration with peer educators, as well as STI screening and treatment, counselling, and testing and care for the HIV infected (Bekker et al., 2015; Das & Horton, 2015; Dhana et al., 2014; Mountain et al., 2014a). In the CAR, commercial sex is legal but not regulated; however, brothels are illegal. Thus, efforts to support progressive policy and legislation concerning FSW through the minimum packages of services for the country will likely encourage decreasing barriers to STI prevention and care services for populations that already face societal discrimination. Finally, early HIV testing, which has been proved to be effective in Bangui to obtain changes of at-risk behaviour (Grésenguet et al., 2002) will allow early antiretroviral treatment with a possible impact on HIV heterosexual transmission, at least for the clients (Low et al., 2015). The final aim is to effect national intervention allowing the expansion and ramping up of both community-based and clinic-based HIV and STI prevention activities for FSWs country-wide. The intervention will use a phased approach with new clinic sites providing services for FSWs. At each site, comprehensive minimum packages of prevention and care services will be offered. Key indicators of the intervention’s success could include an increase in condom use and a decrease of STI/HIV prevalence among the targeted populations.

The poorest and least educated categories are the katas and street vendors, which are also those who consume more alcohol and psycho-active substances (cannabis, tramadol and glue) and, therefore, are the least protected. In a first approach, public health priority could focus prevention efforts on these two categories which are not so distinct from each other. This is mainly to avoid giving leadership to other categories, classically described by UNAIDS as *key populations* (UNAIDS, 2011), among which we find girls who speak or write French better and who are better interlocutors for professional health services. Furthermore, it could be relevant to cross-reference the different groups and subgroups of FSWs and their risk of other STIs such as syphilis and herpes simplex virus infection as important factors of comorbidity in the CAR.

Furthermore, the vulnerable population of FSWs younger than 18 years of age (27% of the studied FSW population) needs specific health interventions, including access to sexual and reproductive health care and rights, and HIV treatment, prevention and care, despite legal and policy barriers and the frequent lack of confidential, adolescent-friendly HIV services, as previously pointed out in Zimbabwe (Busza et al., 2016).
